# Fellow-Workers and the Roll of Honour

**Published:** 1915-05-15

**Authors:** 


### ROUND THE WORLD.

[The Editor invites Early Information and Contributions Marked "Fellow-Workers."]

### Fellow-Workers and the Roll of Honour.

Amongst the Dardanelles casualties one notes with regret the death of Fleet Surgeon Adrian Andrew Forrester, a graduate of Glasgow University, who qualified in 1897, and entered the Royal Navy in the following year. At the outbreak of war the late Mr. Forrester held the rank of Staff surgeon, but he received promotion at the end of last year. He met his death during military operations carried out on April 25.

Surgeon-General M. W. Russell has been calling the attention of hospital officers to the importance of preserving all documentary records of wounded men passing through their hands. In the rush of routine work such things as temperature charts, case-notes, clinical reports, and so forth may very readily be mislaid or not completed where no particular interest attaches; yet when the time comes for analysing present experiences records that now seem unimportant may well be useful to the investigator. Now that arrangements are being made to compile a medical history of the war, the humblest note-taker may at any time be writing notes that will be of the utmost value in the future.

Interesting observations have been made by Sir Thomas Oliver, of Newcastle-on-Tyne, in regard to the way recruits representing different social spheres stand the strain of training. In a report communicated to the *Lancet* recently (April 24, 1915) he points out that men whose occupations entail continuous heavy labour are much more liable to break down in the forties than those who have not had to work so hard. He thinks that many men over forty who suffer when put to military training could keep going were it possible to train them more gradually. Sir Thomas Oliver, who is an M.D. Glasg., F.R.C.P. Lond., and a consulting physician to the Royal Victoria Infirmary at Newcastle, has had ample opportunities of studying these matters in regard to the "Tyneside Scottish," of which he is honorary colonel.

Dr. Ernest Rivaz Hunt, who has been appointed honorary physician to the Brighton, Hove, and Preston Red Cross Hospital, is a Cambridge and St. Mary's man who did well in his student days, and after serving in the "house" at his hospital, where he was for a time house surgeon to Mr. A. J. Pepper, settled in practice at Brighton. After qualifying L.R.C.P., M.R.C.S., he became an M.D. Cantab., and more recently was admitted to the membership of the Royal College of Physicians. He has carried out original observations on the causation and clinical appearances of subphrenic abscess.

A good deal of assistance has been rendered to the French Red Cross by the Comité de Londres, which, under the direction of Mr. James Donelan, has arranged for surgeons to take short periods of duty in the hospitals abroad. There are not a few surgeons who, whilst unable to offer their services for the duration of the war, are glad of an opportunity to work for a month or two under the French Red Cross. The Comité de Londres pays travelling expenses and provides board and accommodation; its headquarters are at 9 Knightsbridge, S.W. Dr. James Donelan is physician in charge of the Nose and Throat Department at the Italian Hospital in Queen Square, and was in his earlier years assistant to the late Sir Morell Mackenzie. He graduated M.B., M.Ch. in Dublin in 1886, and for his services to the Italian community in London was made a Chevalier of the Crown of Italy some years ago.

As a military hospital the Edmonton Infirmary will remain under the command of its present medical superintendent, Dr. Spencer Mort, and it is understood that he will rank as lieutenant-colonel whilst carrying out his new duties. A graduate of Glasgow University, he qualified in 1903, scoring first-class honours and the Brunton prize at the M.B., Ch.B. examination. Dr. Mort became an M.D. Glasg., F.R.C.S. Edin. as recently as 1913. His academic successes also include the William Cullen medal in medicine, the John Hunter medal in surgery, and the Rainy prize in anatomy. Dr. Mort was for a time a research worker at the Pasteur Institute in Paris.

Among other Poor-Law medical officers who have decided to take up military work for the time being are Mr. Alexander Duncan, of Ballymena, and Dr. Robert T. Herron, of Armagh.

Mr. Alexander Duncan, who holds the posts of medical officer to the Glenwhirry Dispensary and honorary medical officer to the Cottage Hospital at Ballymena, studied medicine at Edinburgh University and Queen's College, Belfast, qualifying by taking the Scottish "triple" diploma in 1894. Subsequently he was house surgeon to the Union Infirmary, Belfast. Mr. Duncan is also adviser in his district to the Australian Government in regard to emigrants.

Dr. Robert Thomas Herron, M.D. Durh., M.R.C.P. Edin., qualified at the Dublin Schools in 1883. At the time of the South African War he went to the Front as a civil surgeon, and returned with the medal and four clasps. He is a well-known figure in the Armagh district, where he holds the appointments of medical officer to the Union Infirmary and Fever Hospital, consulting sanitary officer, and physician to the Macan Blind Asylum. The local Guardians have granted Dr. Herron a year's leave of absence.

A student of St. Thomas's Hospital, Mr. Henry Hilton Monk Gould has died in this country from a severe illness initiated whilst he was working under the Red Cross on the Continent. Proceeding to the hospital from Clare College, Cambridge, he won a University Entrance Scholarship some eighteen months ago, and sat for the "primary fellowship" (R.C.S.) with success before proceeding to his clinical work.

A young medical man who went to the Front as a combatant officer in the Territorials, Captain Howard Tomlin Hunter, lost his life in the fighting round Ypres on April 26. Educated at Durham University, where he qualified M.B., B.S. at the age of twenty-three, the late Captain Hunter had been for some time previously very much interested in Territorial work, having been given a commission in the Northumberland Fusiliers in 1906, his promotion occurring not long after he had become medically qualified. Sad to relate, his brother, Captain George Edward Hunter, serving in the same battalion of the Northumberland Fusiliers, and senior to him by a year, also fell in the action on April 26.

